# Ultra-rare monogenic disorders frequently detected among sex chromosome aneuploidy patients with atypical findings

**DOI:** 10.1038/s10038-024-01312-y

**Published:** 2024-12-19

**Authors:** Kiana Magee, William McGonigle, Rena Pressman, Willa Thorson, Deborah Barbouth, Nicholas A. Borja

**Affiliations:** 1 John P. Hussman Institute for Human Genomics, University of Miami Miller School of Medicine, Miami, FL USA; 2 John T. Macdonald Foundation Department of Human Genetics, University of Miami Miller School of Medicine, Miami, FL USA

**Keywords:** Aneuploidy, Genetic testing

## Abstract

Sex chromosome aneuploidies (SCA) such as Turner, Klinefelter, Jacobs, and Trisomy X syndromes are prevalent genetic disorders with well-established phenotypes. Challenges persist, however, in determining the need for further genetic evaluation in cases of affected individuals exhibiting atypical symptoms. The present study retrospectively examined 54 pediatric patients with an SCA diagnosis at a single institution between January 2015 and December 2023. Twelve patients (22.2%) exhibited a discordant phenotype, of which five were confirmed to have a distinct monogenic disorder, a diagnostic rate of 41.7%. The monogenic conditions identified included DNAH5-related primary ciliary dyskinesia, Burn-McKeown syndrome, Tatton-Brown-Rahman syndrome, SETD1B-related neurodevelopmental disorder, and SET-related disorder. The median age at SCA diagnosis was 3.5 months versus 7.0 years for the second genetic condition, indicating significant diagnostic delays. Our findings highlight the importance of comprehensive genetic evaluation in pediatric patients with SCA who exhibit atypical phenotypes.

Sex chromosome aneuploidies (SCA), including Turner (45,X), Klinefelter (47,XXY), Jacobs (47,XYY), and Trisomy X (47,XXX) syndromes are among the most common genetic disorders, each with an estimated prevalence between 1 in 1000 to 1 in 2000, with their associated phenotypes extensively described in the medical literature [1, 2]. These include the classic manifestations such as tall stature and hypogonadism in Klinefelter syndrome or coarctation of the aorta and premature ovarian failure in Turner syndrome, though cases of SCA with unusual or severe symptomatology have also been reported. The widespread adoption of non-invasive prenatal testing (NIPT), however, has led to a much broader ascertainment of affected individuals, thereby deepening our understanding of the phenotypes commonly associated with these disorders [3]. Challenges remain, however, in determining the need for further genetic evaluation among pediatric patients with SCA exhibiting atypical symptoms.

To better characterize the value of additional genetic testing among patients with SCA, we performed a retrospective chart review of 54 pediatric patients evaluated at an academic medical center between January 2015 and December 2023 and cytogenetically diagnosed with a sex chromosome aneuploidy. Of these, a review of available medical records identified 12 patients (22.2%) with discordant or severe phenotypes, eleven of whom received additional molecular testing. A second genetic syndrome associated with the discordant features was identified in five of these atypical SCA cases, a diagnostic rate of 41.7% (Table 1). Variants in each patient were identified through next-generation sequencing and array comparative genomic hybridization performed by accredited commercial clinical laboratories, Quest Diagnostics (Secaucus, NJ), Ambry Genetics (Aliso Viejo, CA), GeneDx (Gaithersburg, MD), and Invitae (San Francisco, CA), and were classified based on the American College of Medical Genetics standards for sequence variant interpretation [4]. The study was approved by the University of Miami Institutional Research Board (IRB #20081166).

**Table 1:** Summary of the five patients from the sex chromosome aneuploidy cohort who exhibited discordant features and for whom additional testing led to a second genetic diagnosis

Patient	Karyotype	SCA concordant features	SCA discordant features	Additional molecular testing	Molecular findings	Associated diagnosis
1	45,X (birth)	Hypoplastic aortic arch, poor feeding (birth); recurrent ear infections, short stature, delayed puberty	Situs inversus (birth)	Multigene panel (14 yo)	*DNAH5* c.5582A>C (p.Gln1861Pro), likely pathogenic, heterozygous, maternally inherited c.6088T>G (p.Cys2030Gly), likely pathogenic, heterozygous, paternally inherited	Primary ciliary dyskinesia 3, with or without situs inversus (MIM 608644)
2	46,X,psu idic (X)(p11.2) (birth)	Small for gestational age, patent foramen ovale (birth)	Corneal opacities, aural atresia, bilateral cleft lip and palate, anteriorly displaced anus, hypotonia (birth)	Exome sequencing (3 mo)	*TXNL4A* c.-222_189del, likely pathogenic, homozygous, maternally and paternally inherited	Burn-McKeown syndrome (MIM 608572)
3	47,XYY (5 mo)	Hypotonia (birth); Motor and speech delay (1 yo)	Intellectual disability, mitral valve insufficiency, central sleep apnea, overgrowth, macrocephaly, microphallus, joint hypermobility	Chromosomal microarray analysis (9 yo)	arr[GRCh37]2p23.2 (24,710,112-25,987,357)x1	Tatton-Brown-Rahman syndrome (MIM 615879)
4	47,XXX (11 mo)	Motor and speech delay (11 mo)	Oppositional defiant disorder (2 yo), seizure disorder (3 yo), obesity with food seeking behavior (4 yo), ventriculomegaly (4 yo), autism spectrum disorder (5 yo)	Exome sequencing (10 yo)	*SETD1B* c.1A>C (p.M1?), pathogenic, heterozygous, de novo	SETD1B-related neurodevelopmental disorder (MIM 619000)
5	47,XXX (4 yo)	Motor and speech delay, hypotonia (2 yo)	Macrocephaly, brain MRI with gliosis, poor balance (4 yo); premature adrenarche (5 yo)	Exome sequencing (5 yo)	*SET* c.362C>G (p.T121S), likely pathogenic, heterozygous, de novo	SET-related neurodevelopmental disorder (MIM 618106)

The age at testing or onset of clinical findings is included in parenthesis where known

Patient 1 was diagnosed with Turner syndrome in the newborn period after findings of redundant nuchal folds, poor feeding, and coarctation of the aorta. Situs inversus totalis, a finding only previously reported in a single Turner syndrome case, was also observed at this time [5]. When the patient was 14 years of age, multigene panel testing identified she was compound heterozygous for the pathogenic variants in *DNAH5*, associated with primary ciliary dyskinesia 3, with or without situs inversus.

The second patient was prenatally suspected to have Turner syndrome due to an abnormal NIPT result and detection of cleft lip and palate in utero, a finding rarely reported in association with Turner syndrome [6]. At birth, chromosomal analysis confirmed dicentric isochromosome Xp11.22q28, consistent with a variant form of Turner syndrome. Physical exam of the neonate revealed discordant features, including corneal opacities, aural atresia, hypotonia, and anterior displacement of the anus. Whole exome sequencing was pursued at 3 months of life, revealing homozygosity for likely pathogenic variants in *TXNL4A*, causing Burn-McKeown syndrome.

Patient 3 presented with hypotonia at birth and developmental delay in infancy, and chromosome analysis confirmed 47,XYY. Over the ensuing years, he was found to have macrocephaly, intellectual disability, seizures, central sleep apnea, and mitral insufficiency, which prompted a repeat genetic evaluation at 9 years of age. Chromosomal microarray revealed a 1.28 Mb deletion of 2p23.3 encompassing *DNMT3A* and associated with Tatton-Brown-Rahman syndrome.

The fourth patient was evaluated for developmental delay at 11 months of age and chromosome analysis was ordered, revealing Trisomy X syndrome. She was subsequently diagnosed with epilepsy, autism spectrum disorder, and oppositional defiant disorder, initially attributed to her sex chromosome aneuploidy based on the existing literature [7]. Genetics referral at 10 years of age due to her persistent and severe symptomatology led to exome sequencing, which identified a pathogenic variant in *SETD1B* causing SETD1B-related neurodevelopmental disorder.

Patient 5 was evaluated for global developmental delay and hypotonia at age 4, at which point chromosomal microarray was performed and detected 47,XXX. Due to discordant features, including macrocephaly and overgrowth, she was then referred to genetics, where exome sequencing identified SET-related disorder in association with the patient’s phenotype.

Among these five SCA patients with atypical symptoms, the median age at sex chromosome aneuploidy diagnosis was 3.5 months, while the median age at second genetic diagnosis was 7.0 years. This disparity in time to diagnosis likely reflects the widespread use of NIPT, which, when high-risk for an aneuploidy, will prompt chromosome analysis in the neonatal period, if not during pregnancy. Nevertheless, we observed that three of the five patients had a delay of 8 years or greater between the onset of their atypical symptoms and their undergoing additional molecular testing for the condition.

These cases demonstrate that the diagnostic delay may be partly attributable to decades of reports in the medical literature of patients with SCA exhibiting atypical phenotypes. Because these were published at a time when next-generation sequencing was unavailable or more limited, these atypical associations should be interpreted with caution. Moreover, the ultra-rare monogenic disorders identified here illustrate how an expanded understanding of gene-disease associations continues to augment the diagnostic yield of comprehensive genetic testing.

While atypical symptoms in pediatric patients with SCA were present in 22.2% of the cases evaluated at our institution, this may be an overestimate due to ascertainment bias, as complex patients are more likely to be referred to an academic medical center. Nevertheless, because only a minority of our total SCA cohort displayed atypical symptoms, we regard the benefit of comprehensive genetic testing in pediatric SCA patients with concordant phenotypes as uncertain. For those SCA patients with discordant features, however, the 41.7% rate of monogenic disorders argues for the value of exome or genome sequencing in this specific patient population. In fact, the diagnostic yield of additional molecular testing seen here may be an underestimation, as exome or genome sequencing was only performed in 25% of the atypical SCA cases evaluated at our institution. Among those atypical SCA cases without monogenic disorders identified, chromosomal microarray was the most frequently obtained test, pursued in 71.4% of the cases (Table 2). Further molecular evaluation was not pursued for these patients at the discretion of the geneticist, though this may have been influenced by the reduced availability of exome or genome sequencing early in the study period. These limitations related to the retrospective nature of this study highlight the need for prospective analyses to further define the benefit of comprehensive molecular testing in SCA patients.

**Table 2:** Summary of the seven patients from the sex chromosome aneuploidy cohort who exhibited discordant features and for whom no second genetic diagnosis was made

Patient	Karyotype	SCA concordant features	SCA discordant features	Additional molecular testing	Molecular findings
6	47,XXY (birth)	Speech delay (2 yo); Oppositional defiant disorder, ADHD (5 yo)	Diaphragmatic hernia, tetralogy of Fallot (birth); seizures (2 wk); intestinal malrotation (4 mo)	Chromosomal microarray (birth)	Negative
7	47,XXY (14 yo)	Tall stature, ADHD (12 yo), hypogonadism (14 yo)	Arachnodactyly, pectus excavatum, mitral insufficiency	*COL3A1* single gene testing (14 yo)	Negative
8	45,X (prenatal)	Motor and speech delay (1 yo); precocious puberty (2 yo); autism spectrum disorder (8 yo)	Ventricular septal defect (prenatal); absence seizures (2 yo); strabismus (3 yo); schizophrenia (8 yo)	Chromosomal microarray, genome sequencing (10 yo)	Negative
9	47,XXX (birth)	Motor and speech delay (2 yo)	PFO, PDA, ventricular septal defect (5 mo)	None	Not applicable
10	47,XXY (birth)	Motor and speech delay (1 yo)	Atrial septal defect, bilateral hydronephrosis (birth)	Chromosomal microarray (2 mo)	Negative
11	47,XXX (2 yo)	Motor and speech delay (16 mo)	Severe metatarsus adductus (18 mo); arachnodactyly, hypotonia (22 mo)	Chromosomal microarray, Fragile X testing (2 yo)	Negative
12	47,XXY (birth)	None	Bilateral hydronephrosis (1 mo)	Chromosomal microarray (birth)	Negative

The age at testing or onset of clinical findings is included in parenthesis where known

The updated American College of Medical Genetics guidelines now recommend exome or genome sequencing as first-line testing for pediatric patients with congenital anomalies, intellectual disability, and developmental delay [8]. Based on the data herein, we suggest the application of this recommendation for exome or genome sequencing to pediatric patients with SCA and atypical symptoms. Furthermore, we encourage health professionals caring for patients with SCA to not delay referring those with atypical symptoms to medical genetics for consideration of further testing.
